# Stand Fast, Boys

**Published:** 1906-02

**Authors:** 


### Editorialets.

THE GULLIBLE PUBLIC.—"Rattle Snake Pete," Rochester, N. Y., is the latest to offer a sure cure for consumption. He gives the pulsating heart of a rattler. He is exploited in the Washington *Post* January 7th. A woman lecturer at Seattle, Wash., induced most of her audience to swallow a teaspoonful of black sand as a cure for indigestion. They all had to have a doctor to relieve the cramps.

